# Improving CBCT image quality to the CT level using RegGAN in esophageal cancer adaptive radiotherapy

**DOI:** 10.1007/s00066-022-02039-5

**Published:** 2023-01-23

**Authors:** Hao Wang, Xiao Liu, Lingke Kong, Ying Huang, Hua Chen, Xiurui Ma, Yanhua Duan, Yan Shao, Aihui Feng, Zhenjiong Shen, Hengle Gu, Qing Kong, Zhiyong Xu, Yongkang Zhou

**Affiliations:** 1 Department of Radiation Oncology, Shanghai Chest Hospital, Shanghai Jiaotong University, Shanghai, China; 2 Department of Radiotherapy, Shandong Cancer Hospital and Institute, Shandong First Medical University and Shandong Academy of Medical Sciences, Jinan, China; 3 Manteia Tech, Xiamen, China; 4 Institute of Modern Physics, Fudan University, Shanghai, China; 5 Department of Radiation Oncology, Zhongshan Hospital, Fudan University, Shanghai, China

**Keywords:** Deep learning, Diagnostic imaging, Neural networks, computer, Organs at risk, Artifacts

## Abstract

**Objective:**

This study aimed to improve the image quality and CT Hounsfield unit accuracy of daily cone-beam computed tomography (CBCT) using registration generative adversarial networks (RegGAN) and apply synthetic CT (sCT) images to dose calculations in radiotherapy.

**Methods:**

The CBCT/planning CT images of 150 esophageal cancer patients undergoing radiotherapy were used for training (120 patients) and testing (30 patients). An unsupervised deep-learning method, the 2.5D RegGAN model with an adaptively trained registration network, was proposed, through which sCT images were generated. The quality of deep-learning-generated sCT images was quantitatively compared to the reference deformed CT (dCT) image using mean absolute error (MAE), root mean square error (RMSE) of Hounsfield units (HU), and peak signal-to-noise ratio (PSNR). The dose calculation accuracy was further evaluated for esophageal cancer radiotherapy plans, and the same plans were calculated on dCT, CBCT, and sCT images.

**Results:**

The quality of sCT images produced by RegGAN was significantly improved compared to the original CBCT images. ReGAN achieved image quality in the testing patients with MAE sCT vs. CBCT: 43.7 ± 4.8 vs. 80.1 ± 9.1; RMSE sCT vs. CBCT: 67.2 ± 12.4 vs. 124.2 ± 21.8; and PSNR sCT vs. CBCT: 27.9 ± 5.6 vs. 21.3 ± 4.2. The sCT images generated by the RegGAN model showed superior accuracy on dose calculation, with higher gamma passing rates (93.3 ± 4.4, 90.4 ± 5.2, and 84.3 ± 6.6) compared to original CBCT images (89.6 ± 5.7, 85.7 ± 6.9, and 72.5 ± 12.5) under the criteria of 3 mm/3%, 2 mm/2%, and 1 mm/1%, respectively.

**Conclusion:**

The proposed deep-learning RegGAN model seems promising for generation of high-quality sCT images from stand-alone thoracic CBCT images in an efficient way and thus has the potential to support CBCT-based esophageal cancer adaptive radiotherapy.

In the initial stage of radiotherapy, a high-quality planning CT scan is obtained and the tumor and various organs at risk (OAR) are delineated by experienced radiation oncologists. The planning CT is then used to design radiotherapy plans. In image-guided radiotherapy for esophageal cancer, a low-quality cone-beam CT (CBCT) scan acquired weekly or daily is used for patient positioning and qualitative assessment of tumor and OARs. A series of scattering and noise artifacts reduce the image quality of CBCT and make it unsuitable for quantitative evaluation (e.g., because of the low soft tissue resolution of CBCT, it is difficult to visually distinguish the boundary between tumor and OARs such as the esophagus and trachea).

Due to the limitation of CBCT image quality, dosimetric advantages derived from MRI-guided adaptive radiotherapy have been confirmed for esophageal cancer in several studies. Boekhoff et al. [1] used T2-weighted phase MRI to find that 27 of 29 patients with esophageal cancer could achieve dose coverage by simulating an online bone-match image-guided radiotherapy treatment for each patient, whereas the other 2 patients needed to be rescheduled to achieve dose coverage due to extreme interfractional changes in esophageal position. Defize et al. [2] also used MRI to discover that nearly 20% and 30% tumor regression occurred in the third and fifth weeks in neoadjuvant radiotherapy and chemotherapy for esophageal cancer patients, and suggested adopting adaptive strategies. Hoffmann et al. [3] proposed that compared with bone registration, soft tissue registration and an adaptive strategy could reduce the tumor expansion boundary by 2–3 mm.

As the shape and position of the tumor and OARs changes during radiotherapy of esophageal cancer, an adaptive strategy driven by CBCT, which has been most widely used in image-guided radiotherapy (IGRT), could also ensure dose coverage of the tumor and further reduce the radiation dose of OARs under the condition that CBCT image quality is greatly improved. In addition, CBCT could prevent OARs from receiving an additional planning imaging dose to a certain extent. However, the image quality of CBCT limits accurate segmentation of tumor and OARs, and inaccurate Hounsfield unit (HU) mapping may also bring uncertainty into the dose calculation, which limits the wide application of CBCT in adaptive radiotherapy, especially for esophageal cancer. Therefore, many studies [4–11] have tried to improve the image quality of CBCT by X‑ray scatter correction using the traditional physical model method, so as to meet the requirements of accurate segmentation and dose calculation in adaptive radiotherapy.

The traditional methods of CBCT image calibration are realized by complex X‑ray scattering simulation (such as software improvement through iterative filtering [4], ray tracing [5], model-based method [6], or Monte Carlo [MC] modeling [7, 8]) or hardware change (such as adding an anti-scattering grid [9], an X‑ray beam blocker with a strip pattern [10], or a lead beam blocker with a lattice shape [11]). These methods are difficult to popularize due to physical model calculation efficiency or hardware limitations.

Instead of trying to fix particular noise artifacts in CBCT images, a more recent line of research using convolutional neural networks (CNNs) attempts to directly generate higher-quality synthetic CT (sCT) from CBCT images by correcting the HU values of CBCT images. This method establishes a complicated mapping between CBCT and CT by training CNNs, thus allowing sCT images to be generated from CBCT directly. sCT have the same anatomic structure as CBCT, and the HU values of tissues are close to those of the reference CT. Many CNN-based architectures have been proposed for image synthesis, the most popular being the U‑net [12–14] and generative adversarial networks (GANs) [15–19], which were used for sCT generation in the current study.

U‑net was adopted mainly because it exploits both global and local features in the image spatial domain, matching the task to suppress global scattering artifacts and local artifacts such as noise in CBCT. In the simplest GAN architecture, two networks compete, including a generator that is trained to obtain synthetic images similar to the input set and a discriminator that is trained to classify whether synthetic images are real or fake, thus improving the generator’s performance. GANs learn a loss that combines both tasks, resulting in realistic sCT images.

At present, two kinds of GAN architectures have mainly been used for sCT generation: the supervised *pix2pix* method [15, 16] and the unsupervised *cycle consistency *method [17–19]. These two methods were originally proposed based on natural image datasets [20–23] and are not ideal for medical images, especially for the thoracic site. The pix2pix method has excellent performance but requires well-paired images aligned at the pixel level, which may not always be available due to respiratory movement and anatomic changes during the scanning gap. The paired images obtained by deformable registration are often used for model training. Although the image quality of sCT has been improved by the pix2pix method, accurate pixel alignment of CBCT-CT images for the thoracic site has not yet been fundamentally solved, and it has been observed that the organ boundary was blurred or discontinuous [16]. The cycle consistency method is not so strict in terms of the training data, and could be effective on paired images with pixel misalignment. Due to no unique solution in the solving process for the unsupervised model, its performance is not the best.

Recently, Kong et al. [24] proposed a new image conversion method called *RegGAN*, aiming at tackling the above problems in medical image-to-image translation. RegGAN regards the misplaced target image as noisy labels, and the image conversion training becomes an unsupervised learning, where the generator is trained with an additional registration network to fit the misaligned noise distribution adaptively. RegGAN aims to search for a common optimal solution to both registration task and image conversion, which makes it a better option for a wide range of scenarios, especially for medical image conversion tasks in which aligned data at the pixel level are not achievable.

The purpose of this study was to improve the image quality of standalone thoracic CBCT to the CT level using RegGAN, in which the registration network was only used for training, while for testing, another deformable registration algorithm was used to generate deformed CT (dCT) as a reference. The similarity of the sCT generated by the RegGAN model and the CBCT was compared with dCT. Dose calculation was completed on synthetic CT, CBCT, and the reference dCT using the same radiotherapy plan to further verify the accuracy of sCT in esophageal cancer adaptive radiotherapy.

## Materials and methods

### Image acquisition and preprocessing

CBCT and planning CT images of 150 patients with esophageal cancer who received radiotherapy under free-breathing conditions were collected in our hospital; 120 pairs as training datasets and 30 pairs as testing datasets. The planning CT images of the patients were acquired using a Siemens CT (Siemens medical systems, Erlangen, Germany) and the CBCT images were scanned by the Varian OBI system equipped with an EDGE linac (Varian, Palo Alto, USA). The scanning and reconstruction parameters of CT and CBCT are shown in Table 1.

**Table 1:** Scanning and reconstruction parameters of CBCT and planning CT

	Tube voltage (kVP)	Tube current (mA)	Spatial resolution (mm^2^)	Slice thickness (mm)	Image size
Planning CT	120	220	0.97 × 0.97	2	512 × 512
CBCT	125	15	0.91 × 0.91	2	512 × 512

*CBCT* cone-beam computed tomography

The CBCT images were used as the reference sequence and the planning CT as the secondary sequence for 3D rigid registration, and then planning CT was served as the ground truth for CBCT training. All CBCT images were resized to the same size, and the excess parts were cut off. The HU values of CBCT and CT were clipped to the range of [−1000, 2000] to prevent the ultra-high HU values of some bones from affecting the training, and then normalized to [−1, 1]. For the testing dataset, a deformable registration was performed on the planning CT to pair it to the corresponding CBCT by a multiresolution B‑spline algorithm, and the deformed CT images were used as a reference to assess the similarity of the generated synthetic CT images.

### Image synthesis with RegGAN

In our question, a pair of misaligned images are equivalent to noisy labels, and the noise is mainly caused by the misalignment of spatial positions; thus, the type of noise distribution is relatively clear and can be expressed as a displacement error generated by a random deformation field which generates random displacement for each pixel. A registration network R based on U‑net is used to correct the result from the generator, and the correction loss is defined as correction loss (*L*_*Corr*_). To evaluate the smoothness of the deformation field, we define the smooth loss (*L*_*Smooth*_) to minimize the gradient of the deformation field. In addition, adversarial loss (*L*_*Adv*_) is added between the generator and the discriminator. The total loss (*L*_*Total*_) is defined as the sum of the three loss functions above. More details about the loss functions are included in the Appendix.

**Fig. 1:**
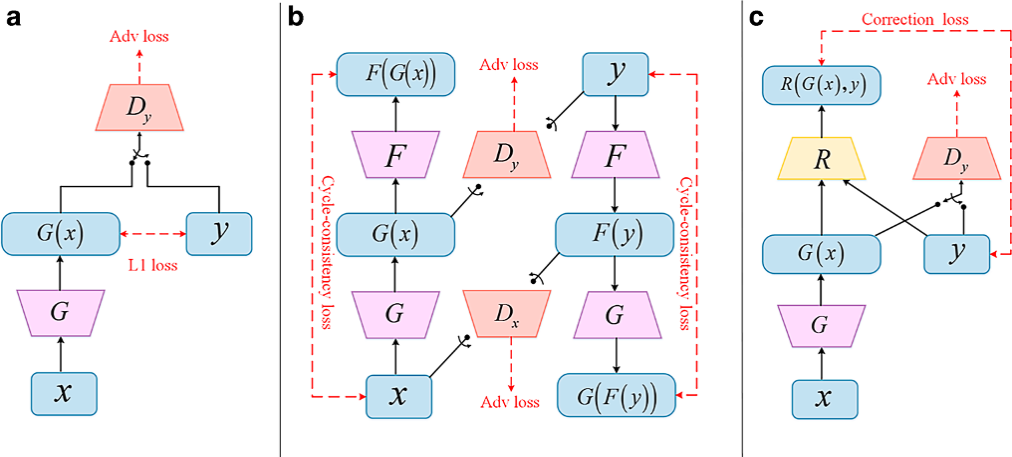
Model diagrams of three kinds of generative adversarial networks: **a** pix2pix, **b** cycle consistency, **c** RegGAN

As shown in Fig. 1c, the loss source of the generator has two parts: one is the adversarial loss conducted by discriminator D, which is the same as in the previous two modes in Fig. 1a,b, and the other is the correction loss between label image y and R (G (x), y) obtained by passing the generated image G (x) through a registration network R. Registration network R is used to correct the noise between x and y caused by spatial positions.

Two down-sampling convolution blocks, nine residual blocks, and two up-sampling de-convolution blocks are used in the generator of the RegGAN model, while four-layer full convolution is designed in the discriminator. Convolution is used to map the input to an N × N matrix. Each point in the N × N matrix represents a small area evaluation value in the original image. The size of the deformation field output from the registration network must be the same as that of the input image. Thus, we use the simplest U‑net structure.

The training conducted on 2D slices was translated and then stacked into 3D volumes; therefore, the network architecture is referred to in this work as 2.5D.

All experiments were carried out in the 64-bit Ubuntu Linux system using PyTorch software (Facebook, Menlo Park, USA), which has 96 GB RAM and 24 GB NVIDIA Titan RTX GPU. All images were normalized to [−1, 1] and then resampled to 512 × 512. All methods were trained using the Adam optimizer with a learning rate of 1e^-4^ and (β1, β2) = (0.5, 0.999). The batch size was set to 1 and the weight attenuation was 1e^-4^. The training process included 50 epochs in total and more than 640,000 iterations. Different weights were also set for different loss functions, as shown in Table 2.

**Table 2:** Loss (*L*) function weight setting

Loss	*L*_*Adv*_	*L*_*Smooth*_	*L*_*Corr*_
Weight	1	10	20

### Evaluation

A side-by-side comparison of dCT, CBCT, and sCT images generated by RegGAN was performed for the testing patients. CBCT and sCT images were quantitatively evaluated by calculating the MAE, RMSE, and PSNR, with deformed CT images as the reference. The metrics such as MAE, RMSE, and PSNR were calculated within the patient outline by an in-house MATLAB script (MATLAB R2016a, MathWorks Inc., MA, USA). MAE measured absolute HU differences of every single pixel between the target and CBCT/sCT image, with lower values indicating closer similarity to the target. RMSE was similar to MAE, which indicated the root of the mean square error. PSNR measured the maximum possible power of a signal, with higher values indicating better image quality.

The HU parameters (mean value, standard deviation, median value, HU integral, HU total count, max HU, and min HU) were acquired from the regions of interest (ROIs; clinical target volume [CTV], planning target volume [PTV], left lung, right ling, total lung, heart, and spinal cord) on each kind of image, which were rigidly copied from the planning CT in the MIM maestro system (MIM Software Inc, USA). These metrics were used to evaluate the similarity of the HU distribution within each ROI on CBCT/sCT images and the reference dCT images. The image quality indices were compared by paired Wilcoxon signed-rank test and the statistical significance level was set at *p* < 0.05.

To verify the dose calculation accuracy, the treatment plans of the 30 testing patients were transferred to dCT, CBCT, and sCT images, and the dose calculation was directly performed without optimization on these images in the Pinnacle 9.10 planning system (Philips Radiation Oncology Systems, Fitchburg, WI). All treatment plans (50.4 Gy/28 fractions) included 4–5 step-and-shoot IMRT fields with 6‑MV X‑rays, and a Varian EDGE linac was used for treatment plans. The dose distribution of the entire treatment plan calculated on deformed CT images was served as reference, the gamma passing rates of the dose distribution calculated on CBCT and sCT images were analyzed using three different criteria (1 mm/1%, 2 mm/2%, and 3 mm/3%).

## Results

### Development of loss values during training

**Fig. 2:**
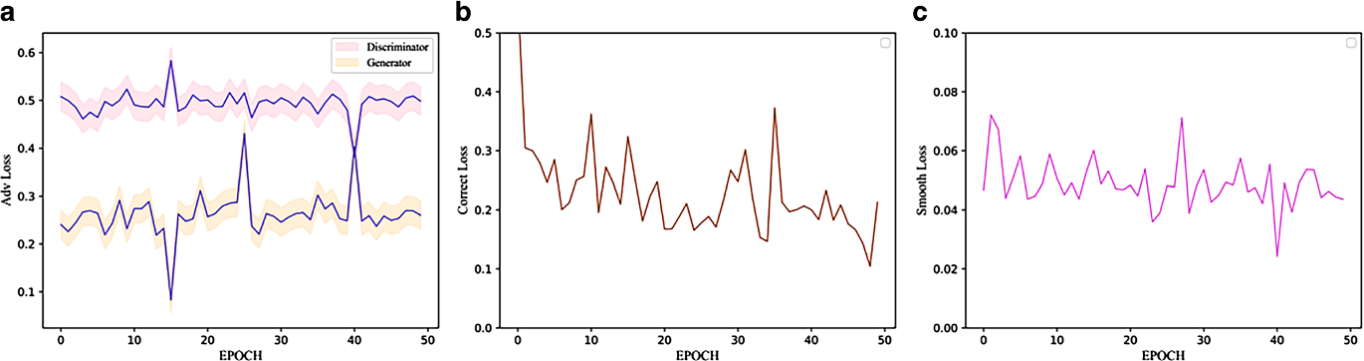
The iterative curves of adversarial loss (**a**), correction loss (**b**), and smooth loss (**c**)

The loss results of each epoch are plotted in Fig. 2. The adversarial loss between the discriminator and the generator is obviously negatively correlated (Fig. 2a), i.e., the more authentic the generated image from the generator is, the more the discriminator cannot distinguish. On the contrary, with a stronger discriminator’s discrimination ability, the loss of the generator will also rise.

The correction loss shows a downward trend as a whole in Fig. 2b, which indicates that the registration network plays a positive role in correcting noise. With the increase in epoch training, the generated sCT becomes closer and closer to the real CT images.

The smooth loss is shown in Fig. 2c. Generally speaking, the stronger the noise, the stronger the noise correction ability required by the registration network, i.e., the lower the smoothness. We can see that the smooth loss has a certain value from beginning to end, which indicates that there is a certain noise phenomenon in the training data of medical images.

All experiments were implemented in Pytorch software in a 64-bit Ubuntu Linux system with 96 GB RAM and 24 GB Nvidia Titan RTX GPU. As the generated CT images are synthesized from 2D images and the network structure is relatively simple, the training and testing speed will not be too slow. Under the conditions of 50 epochs, the training duration is about 6 h. In the actual test, the translation for one patient was completed in about 15 s.

### Comparison of sCT and CBCT images

**Fig. 3:**
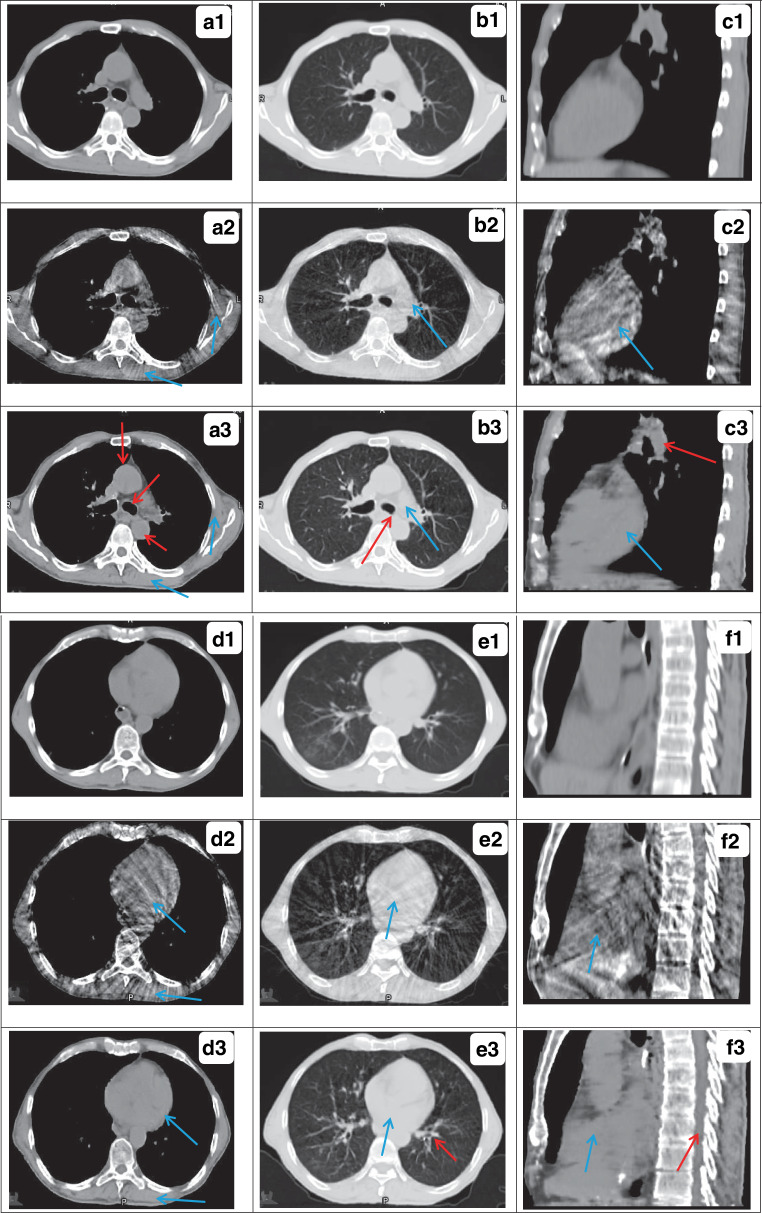
Quality comparison of deformed CT (dCT), cone-bean CT (CBCT), and synthetic CT (sCT) images generated by RegGAN for the same patient in axial or sagittal images. The image types are marked with numbers: *1* stands for dCT, *2* for CBCT and *3* for sCT. Respectively, in the upper right corner of the image, the same axial or sagittal planes are labeled as **a**, **b**, **c**, **d**, **e**, and **f** in turn. *Blue arrows* serious artifacts, *red arrows* good tissue continuity. The display window in second the column is [−1200 300] Hounsfield units (HU; lung window), and the display window in other rows is [−400 400] HU

The sCT images generated from CBCT by the RegGAN model are shown in Fig. 3 for the same testing patient. Serious shading and streaking artifacts were observed at the chest wall, heart, lung, and other sites of the original CBCT images (blue arrows in a2, b2, c2, d2, e2, and f2), due to the influence of patients’ respiratory movement during scanning. The lung window shows that serious distortion occurred in the lung HU value in CBCT, which makes lung seem relatively dark. Most of the artifacts in the sCT images were eliminated (blue arrows shown in a3, b3, c3, d3, e3, and f3), and the anatomic structure was well maintained compared to the original CBCT images (red arrows in a3, b3, c3, and e3). Good tissue continuity of small-volume tissues such as the spinal cord was also revealed in the sagittal images (red arrow in f3).

The results in the testing dataset compared to several previous studies [12–14] on thoracic sites are summarized in Table 3. sCTs generated by deep-learning-based RegGAN showed improved image quality with fewer discrepancies (smaller MAE) to reference dCTs. The results of the proposed model had a similar performance in terms of the improvement of sCT image quality compared to the results of Gao et al. [13] and Qiu et al. [14]. The mean MAE was improved from 80.1 ± 9.1 HU (CBCT vs. dCT) to 43.7 ± 4.8 HU (sCT vs. dCT), and the PSNR also increased significantly from 21.3 ± 4.2 (CBCT vs. dCT) to 27.9 ± 5.6 (sCT vs. dCT) in the testing dataset. In addition, the mean RMSE was improved from 124.2 ± 21.8 HU (CBCT vs. dCT) to 67.2 ± 12.4 HU (sCT vs. dCT).

**Table 3:** Comparison of several studies on the improvement of sCT image quality

	MAE	RMSE	PSNR
CBCT^a^	80.1 ± 9.1	124.2 ± 21.8	21.3 ± 4.2
sCT (RegGAN^a^)	43.7 ± 4.8	67.2 ± 12.4	27.9 ± 5.6
CBCT [Gao] [16]	92.8 ± 16.7	/	21.6 ± 2.8
sCT (pix2pix) [Gao] [16]	53.4 ± 9.3	/	26.8 ± 2.7
sCT (cycleGAN) [Gao] [16]	47.1 ± 6.5	/	28.3 ± 2.0
sCT (AGGAN) [Gao] [16]	43.5 ± 6.7	/	29.5 ± 2.4
CBCT [Qiu] [19]	110.0 ± 24.9	/	23.0 ± 4.0
sCT (cycleGAN) [Qiu] [19]	82.0 ± 17.3	/	28.3 ± 6.9
sCT (cycleGAN + Perceptual + MaxInfo) [Qiu] [19]	66.2 ± 8.2	/	30.3 ± 6.1
CBCT [N Dahiya] [15]	162.8 ± 53.9	328.2 ± 84.7	22.2 ± 2.4
sCT (pix2pix) [N Dahiya] [15]	43.6 ± 22.7	102.8 ± 42.9	32.8 ± 3.8

*MAE* mean average error, *RMSE* root mean square error, *PSNR* peak signal-to-noise ratio

^a^results from the current study

**Fig. 4:**
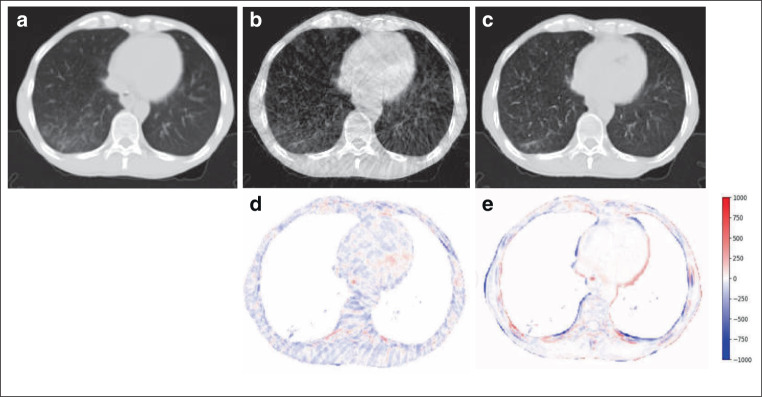
Results of residual images: **a** deformed CT (dCT), **b** cone-beam CT (CBCT), **c** synthetic CT (sCT), **d** residual image between CBCT and deformed CT, **e** residual image between synthetic CT and deformed CT; the generated sCT showed much closer Hounsfield units to the reference dCT

Residual images derived from CBCT/sCT minus dCT are shown in Fig. 4 for one patient from the testing dataset. This shows that sCT image quality was improved, with HU much closer to the reference dCT. Fig. 4a–c show the same axial slice of the lung window display and Fig. 4d and e show the HU difference images between CBCT, sCT, and the reference dCT. Fig. 4a, b, and c display the reference dCT, CBCT, and sCT images, respectively. Serious streaking and shading artifacts could be observed at the heart, chest wall, and bone in the original CBCT images, due to the influence of patients’ respiratory movement during CBCT scanning. Most of the artifacts on the sCT images were eliminated, especially at the heart and chest wall. However, there seems to be quite large error in the sCT at the tissue interfaces, which may essentially be caused by the small alignment error between the CBCT and the real CT.

The HU parameters mean value, median value, HU integral, and HU total count are much closer between sCT and the reference dCT compared to CBCT and dCT within the ROIs. The HU parameters mean value, median value, HU integral, and HU total count of CBCT and sCT images are similar to those of dCT images within the ROIs of left lung, right lung, and total lung, and these parameters of sCT images seem to be closer to those of dCT images compared to CBCT images (more details are shown in Table 6 in the Appendix).

**Fig. 5:**
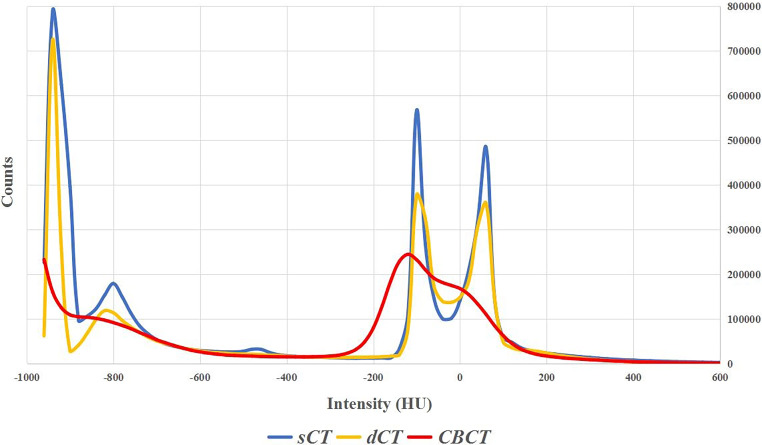
Histogram distribution curves of the Hounsfield unit (*HU*) values of deformed CT (*dCT*), cone-beam CT (*CBCT*), and synthetic CT (*sCT*) images from one test patient

The HU value and the number of occurrences of HU values are denoted for one test patient in Fig. 5. The HU value distributions of the CBCT and reference dCT images clearly differed, while the sCT images generated by RegGAN showed a similar HU distribution to the dCT images. Due to the large volume of total lung, there was an obvious peak at about −900 on the HU distribution of dCT and sCT images, but this was not the case in the HU distribution of CBCT images.

### Dose calculation

**Fig. 6:**
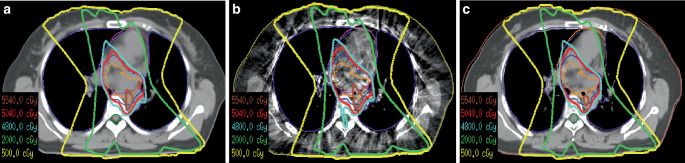
The isodose lines shown on the same transverse slice of (**a**) deformed CT (*dCT*), (**b**) cone-beam CT (*CBCT*), and (**c**) synthetic CT (*sCT*) calculated by the same treatment plan for one testing patient

The absolute dose distributions calculated on dCT, CBCT, and sCT images by the same treatment plan are shown in Fig. 6 for one test patient. It can be seen that the 48 Gy isodose line displayed on sCT images is much closer to that on dCT, while the 48 Gy isodose line shown on CBCT has significant distortion.

**Fig. 7:**
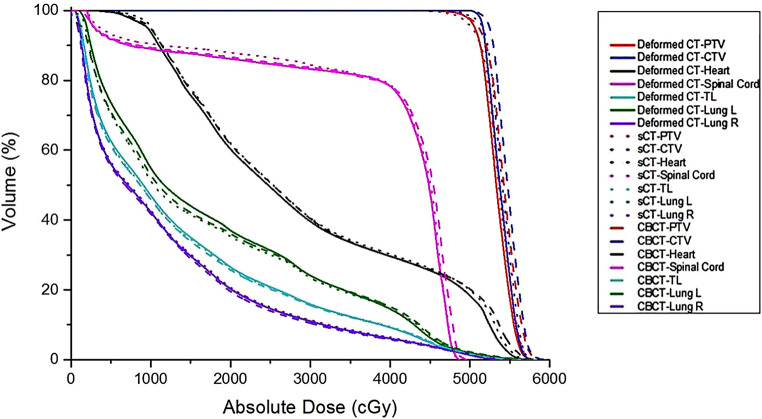
Dose–volume histograms (DVHs) of the same treatment plan calculated on the deformed CT (*dCT*), cone-beam CT (*CBCT*), and synthetic CT (*sCT*). The *solid line* is the DVH calculated on dCT, the *dotted line* is the DVH calculated on sCT, and the *long-dashed line* is the DVH of the CBCT images. *CTV* clinical target volume, *PTV* planning target volume, *Lung L* left ling, *Lung R* right lung, *TL* total lung

Dose–volume histograms (DVHs) of the same treatment plan calculated on the dCT, sCT, and CBCT scans are displayed for the same patient in Fig. 7. DVHs of the CTV, PTV, heart (> 45 Gy), and spinal cord (> 40 Gy) show closer profiles calculated on dCT and sCT images.

DVH parameters for targets and OARs are shown in Table 4, which were calculated on dCT, CBCT, and sCT for 30 testing patients. It can be seen that compared with the DVH parameters calculated on CBCT, the DVH parameters calculated on sCT are much closer to those calculated on dCT.

**Table 4:** Dode–volume histogram parameters of treatment plans calculated on dCT, CBCT, and sCT

	PTV D98	Total lung V5	Total lung V20	MLD	Spinal cord Dmax	Heart V30	MHD
dCT	49.14 ± 0.32	54.17 ± 6.61	21.95 ± 3.15	11.63 ± 1.43	43.32 ± 2.02	27.35 ± 10.38	21.52 ± 6.71
CBCT	47.87 ± 2.48	54.82 ± 6.24	22.13 ± 3.21	11.72 ± 1.42	43.73 ± 1.99	27.92 ± 10.58	21.87 ± 6.88
sCT	48.32 ± 0.97	54.12 ± 6.55	22.12 ± 3.31	11.66 ± 1.42	43.58 ± 2.03	27.18 ± 10.74	21.42 ± 6.89

V5, V20, and V30 represents as percentage volume of 500 cGy, 2000 cGy, 3000 cGy dose coverage

*dCT* deformed CT, *CBCT* cone-beam CT, *sCT* synthetic CT, *PTV* planning target volume,* MLD* mean lung dose, *Dmax* maximum dose, *MHD* mean heart dose

**Fig. 8:**
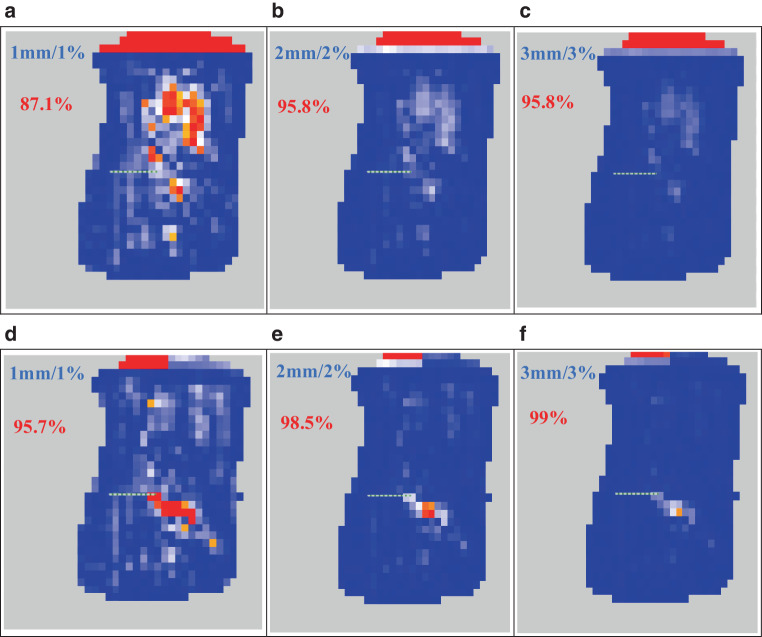
The gamma analysis index distribution calculated on original cone-beam CT (CBCT; **a**, **b**, **c**) and generated synthetic CT (sCT; **d**, **e**, **f**) images, with dose on the deformed CT image as reference using entire intensity-modulated radiation therapy fields for one test patient using three criteria

Using the dose distribution calculated on dCT images as a reference, the absolute gamma analysis distribution of the corresponding CBCT and sCT images under the criteria 3 mm/3%, 2 mm/2%, and 1 mm/1% are shown for one testing patient in Fig. 8. The dose distributions on the original CBCT images remained highly divergent compared with the reference. There are large regions where the gamma index is greater than 1 on CBCT images. The dose distributions on the sCT images are close to the reference, and the areas with a gamma index greater than 1 are greatly reduced.

The statistical analysis of gamma passing rates with different standards for the 30 testing patients are shown in Table 5. The gamma passing rates of sCT images generated from RegGAN were significantly improved under all criteria compared to those of the original CBCT (*p* < 0.05). In conclusion, the sCT images generated by RegGAN obtained more accurate dose calculations in radiotherapy for testing patients.

**Table 5:** The gamma passing rates of dose distribution in CBCT and sCT images for patients

	Gamma criteria		
	1 mm/1%	2 mm/2%	3 mm/3%
CBCT (%)	72.5 ± 12.5	85.7 ± 6.9	89.6 ± 5.7
RegGAN (%)	84.3 ± 6.6	90.4 ± 5.2	93.3 ± 4.4

*CBCT* cone-beam CT, *sCT* synthetic CT

## Discussion

Weekly and daily CBCT images are currently only used for patient positioning during IGRT. Due to poor HU mapping and low soft tissue contrast due to more obvious motion artifacts caused by respiratory movement at the thoracic site, CBCT images are not suitable for esophageal cancer adaptive radiotherapy at present.

Compared with the traditional complex physical model used to simulate X‑ray scattering or hardware modification to improve the quality of CBCT images, several kinds of GAN model based on deep learning to improve the quality of CBCT images have been reported for their high efficiency and feasibility, especially for the thoracic site [15–19]. As can be seen from the current results, the image quality as improved by GAN models was not only determined by the image quality of the training data, but also by the method of image pre-processing, the model framework, and parameter settings.

As the image quality of CBCT for the thoracic site is affected by even more uncertain factors, whether it can be effectively improved seems to have become a stress test to verify the efficiency and accuracy of the image conversion models based on deep learning. Several studies [15, 16] used supervised learning methods such as the pix2pix method to generate synthetic images of the thoracic site, which significantly reduced artifacts and improved soft tissue resolution on sCT images. As the pixel-alignment accuracy of paired CBCT-CT images was mainly affected by deformable registration, it is also observed from these results that the anatomic structures on the sagittal/coronal plane of the generated synthetic images were discontinuous or the segmentation of small organ structures was inaccurate. In short, pix2pix can only generate high-quality sCT images under the premise of accurate alignment between CBCT and CT images.

For unsupervised learning methods, such as cycleGAN-type methods, generating sCT for the thoracic site [17, 18], it has been demonstrated that the generated sCT could maintain the anatomic structures well, and sagittal/coronal images have continuous structures due to the non-necessity of CBCT-CT deformable registration, while several artifacts may get propagated into the final sCT images. As thoracic CBCT images usually have more artifacts due to patients’ respiratory motion, it is difficult to inhibit serious artifacts, especially at the chest wall and heart for cycleGAN-type methods. Qiu et al. [19] proposed the cycleGAN method combined with histogram matching; perceptual supervision was adopted to minimize blurring of tissue interfaces by using paired planning CT and deformed CBCT.

We also found that sCT looks more consistent with the reference in the axial plane than in the sagittal plane, which may be because the training we conducted was on 2D slices in the RegGAN model. It could also be observed that greater error occurred at the tissue interfaces (Fig. 4), which could be explained by the fact that when CBCT is converted to sCT, HU will approach the HU of real CT (HU becomes larger), while in fact, the original CBCT image cannot be perfectly aligned with the real CT. In the area with small alignment error, a larger error of HU values which is not caused by CT synthesis will appear at the tissue interfaces between CBCT and the real CT.

This study proposes a new image translation model (RegGAN). RegGAN regards the misplaced target image as noisy labels and transforms the image conversion training into an unsupervised learning process with noisy labels. The paired images trained in RegGAN do not need deformable registration, which could be adaptively compensated by a well-trained registration network to fit the misaligned noise distribution. RegGAN could find a common optimal solution to both the registration network and image conversion, which could effectively reduce the unpredictable changes of anatomic tissue position on sCT images while maintaining the image quality of the original planning CT. The sCT images have clear and continuous anatomic structure boundaries in the sagittal and coronal planes, especially for small-volume anatomic structures such as esophagus and spinal cord.

In general, 4D-CBCTs showed considerably lower image quality than 3D-CBCTs. This difference can be attributed to the low number of projections available for a single phase of the 4D-CBCT. Thummerer A [25] verified the feasibility of deep-learning-based 4D sCTs from sparse-view CBCTs for dose calculations in adaptive proton therapy. In addition, MR-guided radiotherapy treatment planning utilizes the high soft tissue contrast of MRI to reduce uncertainty in delineation of the target and organs at risk. Replacing 4D-CT with MRI-derived synthetic 4D-CT would support treatment plan adaptation on hybrid MR-guided radiotherapy systems for inter- and intrafractional differences in anatomy and respiration [26, 27]. We mainly focus on using deep learning to decrease CBCT image noise, acquired in CBCT imaging, which belongs to estimating the noise transition matrix, and simplify the problem to obtain a prior distribution of noise. CBCT image quality can be significantly improved in the image-to-image translation using a registration network, and this work can also be transferred to 4D sCT (MR) image translation.

For the unpaired training images, no additional manual deformation registration is required, which could be resolved by the combination of the registration network and GAN approach, and further reduce the labor cost. As we synthesize from 2D images and the network structure is relatively simple, the training and testing speed will not be too slow. Under the conditions of 50 epochs, the training duration is about 6 h. In the actual test, the translation for one patient was completed in about 15 s. RegGAN may be an effective option for a wide range of scenarios, especially for the thoracic site for which aligned data at the pixel level are not achievable.

## Conclusion

Unpaired thoracic CBCT and CT images were trained by RegGAN. The synthetic high-quality CT images maintained the anatomic structures well and reduced most artifacts. The sCT generated by RegGAN provided high-accuracy dose calculation and can thus be applied to esophageal cancer adaptive radiotherapy.

## Appendix

### Model details

A given training dataset ${\left\{\left(x_{n}{,}\tilde {y}_{n}\right)\right\}}_{n=1}^{N}$ has *n* noise labels, where $x_{n}{,}\tilde {y}_{n}$ are images from two modes, and it is assumed that *y*_*n*_ is a clean label of *x*_*n*_, but is unknown. In the problem setting, the type of noise distribution is clear, which could be expressed as displacement error: $\tilde {y}=yo T$, where, *T* is represented as a random deformation field which causes each pixel to have a random displacement. Therefore, a registration network can be connected after the generator *G* as a noise model *R* to correct the results of the generator *G*(*x*). Thus, the correction loss is obtained:

1 $$\min _{G{,}R} \ L_{\mathrm{Corr}}\left(G{,}R\right)=E_{x{,}\tilde {y}}\left[\left|\left|\tilde {y}-G\left(x\right)o R\left(G\left(x\right){,}\tilde {y}\right)\right|\right|_{1}\right]$$

$R\left(G\left(x\right){,}\tilde {y}\right)$ represents the deformation field derived from *G*(*x*), $\tilde {y}$ registration for fitting *T*, and E represents the expectation of the function. Therefore, we also need to calculate a smooth loss on the deformation field to minimize the gradient of the deformation field to make the deformation as smooth as possible:

2 $$\min _{R} \ L_{\text{Smooth}}\left(R\right)=E_{x{,}\tilde {y}}\left[\Updelta \left|\left|R\left(G\left(x\right){,}\tilde {y}\right)\right|\right|^{2}\right]$$

Finally, add the adversarial loss of the generator and the discriminator to form the whole loss:

3 $$\min _{G}\ \max _{D}\ L_{\mathrm{Adv}}\left(G{,}D\right)=E_{y}\left[\log \left(D\left(y\right)\right)\right]+E_{x}\left[\log \left(1-D\left(G\left(x\right)\right)\right)\right]$$

4 $${\min _{G{,}R}\ \max _{D}\ {L_{\text{Total}}}\left(G{,}R{,}D\right)={L_{\mathrm{Corr}}}+{L_{\text{Smooth}}}+{L_{\mathrm{Adv}}}}$$

**Table 6:** HU parameters of ROIs on dCT, CBCT, and sCT images for testing patients

HU parameter		CTV	PTV	Left lung	Right lung	Total lung	Heart	Spinal cord
Mean value	*dCT*	−16.7 ± 61.3	−30.6 ± 47.9	−668.5 ± 73.7	−687.9 ± 56.8	−678.9 ± 64.0	19.7 ± 10.4	22.2 ± 7.1
	*CBCT*	−42.5 ± 76.6	−57.5 ± 59.7	−701.6 ± 85.9	−719.9 ± 78.9	−712.5 ± 81.7	−13.6 ± 17.9	5.3 ± 9.6
	*sCT*	−14.7 ± 60.1	−31.9 ± 48.2	−650.9 ± 78.1	−661.8 ± 86.3	−657.1 ± 82.7	17.7 ± 7.4	31.1 ± 5.7
Standard deviation	*dCT*	163.5 ± 74.5	205.9 ± 55.3	202.7 ± 11.3	191.8 ± 4.5	198.7 ± 6.1	57.9 ± 27.5	49.1 ± 13.1
	*CBCT*	188.9 ± 64.9	227.2 ± 52.5	224.0 ± 12.7	205.6 ± 11.4	213.4 ± 10.8	97.7 ± 20.8	92.3 ± 8.9
	*sCT*	174.3 ± 72.4	214.3 ± 56.9	221.4 ± 20.9	220.7 ± 17.6	221.7 ± 15.0	71.4 ± 23.1	60.2 ± 15.6
Median value	*dCT*	28.7 ± 20.6	31.0 ± 13.4	−741.2 ± 74.7	−761.5 ± 59.8	−752.7 ± 65.7	37.3 ± 2.1	18.8 ± 3.3
	*CBCT*	−4.3 ± 43.2	−6.0 ± 34.3	−760.8 ± 93.6	−774.8 ± 85.4	−768.8 ± 88.6	−5.0 ± 9.9	3.2 ± 11.3
	*sCT*	34.7 ± 18.4	35.2 ± 14.3	−732.2 ± 79.2	−747.3 ± 82.6	−741.0 ± 81.3	39.7 ± 3.4	22.5 ± 3.1
HU integral^a^	*dCT*	−2.2*10^3^ ± 8.1*10^3^	−8.7*10^3^ ± 1.4*10^4^	−7.1*10^5^ ± 3.7*10^5^	−1.1*10^6^ ± 4.7*10^5^	−1.8*10^6^ ± 8.2*10^5^	1.2*10^4^ ± 7.5*10^3^	5.2*10^2^ ± 1.9*10^2^
	*CBCT*	−5.4*10^3^ ± 1.0*10^4^	−1.6*10^4^ ± 1.7*10^4^	−7.5*10^5^ ± 4.0*10^5^	−1.1*10^6^ ± 5.1*10^5^	−1.9*10^6^ ± 8.8*10^5^	−7.1*10^3^ ± 8.8*10^3^	1.1*10^2^ ± 2.0*10^2^
	*sCT*	−1.9*10^3^ ± 8.0*10^3^	−9.1*10^3^ ± 1.4*10^4^	−7.0*10^5^ ± 3.8*10^5^	−1.0*10^6^ ± 4.9*10^5^	−1.7*10^6^ ± 8.4*10^5^	1.1*10^4^ ± 6.1*10^3^	7.0*10^2^ ± 9.9*10
HU total count	*dCT*	−1.3*10^6^ ± 4.9*10^6^	−5.3*10^6^ ± 8.5*10^6^	−4.4*10^8^ ± 2.3*10^8^	−6.5*10^8^ ± 2.8*10^8^	−1.1*10^9^ ± 5.0*10^8^	7.3*10^6^ ± 4.6*10^6^	3.2*10^5^ ± 1.2*10^5^
	*CBCT*	−3.3*10^6^ ± 6.2*10^6^	−1.0*10^7^ ± 1.1*10^7^	−4.6*10^8^ ± 2.4*10^8^	−6.9*10^8^ ± 3.1*10^8^	−1.1*10^9^ ± 5.4*10^8^	−4.3*10^6^ ± 5.3*10^6^	6.6*10^4^ ± 1.2*10^5^
	*sCT*	−1.2*10^6^ ± 4.9*10^6^	−5.5*10^6^ ± 8.6*10^6^	−4.3*10^8^ ± 2.3*10^8^	−6.4*10^8^ ± 3.0*10^8^	−1.1*10^9^ ± 5.2*10^8^	6.4*10^6^ ± 3.7*10^6^	4.3*10^5^ ± 6.0*10^4^
Max value	*dCT*	493.8 ± 78.7	703.8 ± 170.7	651.2 ± 111.7	725.2 ± 202.5	787.0 ± 147.2	264.7 ± 86.9	642.2 ± 129.9
	*CBCT*	690.7 ± 103.0	1062.0 ± 237.9	1067.2 ± 222.1	1021.7 ± 158.5	1117.3 ± 200.5	485.7 ± 119.6	956.0 ± 169.3
	*sCT*	626.3 ± 197.8	847.3 ± 344.1	782.0 ± 221.1	865.2 ± 150.9	919.8 ± 172.9	380.0 ± 210.0	914.7 ± 106.5
Min value	*dCT*	−999 ± 1.5	−1000.0 ± 0.0	−653.2 ± 808.4	−998.8 ± 2.0	−999.3 ± 0.8	−705.5 ± 383.6	−90.0 ± 131.0
	*CBCT*	−1000.0 ± 6.0	−1000.0 ± 0.0	−1000.0 ± 0.0	−1000.0 ± 0.0	−1000.0 ± 0.0	−917.0 ± 176.5	−338.3 ± 56.3
	*sCT*	−1008.2 ± 10.6	−1015.3 ± 7.7	−1011.8 ± 29.0	−1017.3 ± 39.9	−1022.3 ± 37.0	−917.7 ± 90.1	−176.5 ± 44.3

*HU *Hounsfield units, *dCT* deformed CT, *CBCT* cone-beam CT, *sCT* synthetic CT, *CTV *clinical target volume, *PTV* planning target volume, *ROIs* regions of interest

^a^HU integral represents the cumulative sum of HU values in the volume of the region of interest; units are HU * cm^3^; units of other parameters in the table are HU
